# The Positional Relationship Between the Mandibular Canal and the Lower Third Molar Determined on Cone-Beam Computed Tomography

**DOI:** 10.3390/medicina61071291

**Published:** 2025-07-17

**Authors:** Horatiu Urechescu, Ancuta Banu, Marius Pricop, Felicia Streian, Alisia Pricop, Cristiana Cuzic

**Affiliations:** 1 Department of Oral and Maxillo-Facial Surgery, Faculty of Dental Medicine, University of Medicine and Pharmacy “Victor Babes”, 300041 Timisoara, Romania; urechescu.horatiu@umft.ro (H.U.); streian.felicia@umft.ro (F.S.); 2 Research Center in Dental Medicine Using Conventional and Alternative Technologies, School of Dental Medicine, University of Medicine and Pharmacy “Victor Babes”, 300041 Timisoara, Romania; alisia.pricop@umft.ro; 3 Department of Prosthodontics, Faculty of Dental Medicine, University of Medicine and Pharmacy “Victor Babes”, 300041 Timisoara, Romania; pricop.cristiana@umft.ro; 4 TADERP Research Center—Advanced and Digital Techniques for Endodontic, Restorative and Prosthetic Treatment, University of Medicine and Pharmacy “Victor Babes”, 300041 Timisoara, Romania

**Keywords:** third molar, mandibular canal, CBCT

## Abstract

*Background and Objectives*: The extraction of mandibular third molars poses challenges due to their proximity to the mandibular canal and risk of inferior alveolar nerve (IAN) injury. Accurate preoperative evaluation is essential to minimize complications. This study assessed the three-dimensional positional relationship between the mandibular canal and lower third molars using cone-beam computed tomography (CBCT), aiming to identify anatomical positions associated with increased surgical risk. *Materials and Methods*: This retrospective study analyzed 253 CBCT scans of fully developed lower third molars. The mandibular canal position was classified as apical (Class I), buccal (Class II), lingual (Class III), or interradicular (Class IV). Contact was categorized as no contact, contact with a complete or defective white line, or canal penetration. In no-contact cases, the apex–canal distance was measured. Statistical analysis included descriptive and contingency analyses using the Chi-Square Likelihood Ratio test. *Results*: Class I was most common (70.8%) and presented the lowest risk, while Classes III and IV showed significantly higher frequencies of canal contact or penetration. Class II exhibited shorter distances even in no-contact cases, suggesting residual risk. Statistically significant associations were found between canal position and both contact type (*p* < 0.001) and apex–canal distance (*p* = 0.046). *Conclusions*: CBCT offers valuable insight into the anatomical relationship between third molars and the mandibular canal. High-risk positions—particularly lingual and interradicular—require careful assessment. Even in the absence of contact, close proximity may pose a risk and should inform surgical planning.

## 1. Introduction

Mandibular third molar extraction remains a significant challenge in dental practice. Its anatomical relationship with the mandibular canal plays a critical role in treatment planning and the prevention of complications. Studies on third molar development have shown that factors such as patient age, changes in the structure and position of the mandibular canal, the developmental stage of the third molar, and its position during eruption are crucial in formulating an appropriate surgical plan [1].

Understanding the developmental stages of the third molar is essential for evaluating the risks associated with its proximity to the mandibular canal. The second and third molars are connected to their respective tooth germs via the “gubernaculum dentis,” a structure that influences their development and positioning [2]. During the eruption phase, the spatial relationship between the roots of the third molar and the mandibular canal can shift significantly. Research indicates that in younger individuals with partially erupted third molars or incompletely developed roots, the risk of complications is lower compared to older patients. In the latter group, fully developed roots tend to be in closer proximity to the mandibular canal, increasing the risk of injury [3].

Third molars typically erupt between the ages of 17 and 21, though development and full eruption may continue until age 25 or later [4]. While no major racial differences have been reported in eruption timing, anthropological and dental studies suggest that genetic and environmental factors contribute to certain variations [5,6]. The causes of impacted or unerupted third molars are multifactorial and only partially understood. Common contributing factors include craniofacial development, abnormal positioning of the tooth germ, insufficient eruption space, and failure of bone resorption due to local or systemic conditions [7].

Morphometric analyses have shown that the size of the mandibular angle, which correlates with facial typology and mandibular growth patterns, affects the position and eruption of the mandibular third molar. A larger mandibular angle, typical of the leptoprosopic facial type, is often associated with third molars positioned farther from the mandibular canal. In contrast, a smaller mandibular angle, characteristic of the euryprosopic facial type, results in closer proximity. Even when sufficient space is available, excessive angulation of the third molar can still lead to impaction [8].

Additionally, the thickness and height of the horizontal ramus of the mandible are important in determining both the position of the third molar and its distance from the mandibular canal. Generally, a thicker horizontal ramus correlates with a greater distance between the third molar and the canal, potentially lowering the risk of injury to the inferior alveolar nerve during extraction. These anatomical characteristics are particularly relevant in patients with smaller mandibular angles, where the horizontal ramus tends to be wider and more robust, favoring safer molar positioning. Conversely, in individuals with larger mandibular angles and thinner rami, the molars are typically closer to the canal, thereby increasing the risk of nerve damage.

Another important factor associated with the risk of inferior alveolar nerve injury is the position of the mandibular canal in relation to the third molar—whether apical, buccal, lingual, or interradicular. A higher risk is observed when the canal lies lingual to the molar roots [9].

Given these risks, identifying the most effective imaging modalities to support optimal treatment planning has been a primary focus of research. Owing to their accessibility, orthopantomographs (OPGs) remain the most widely used initial radiographic examination for assessing the relationship between the third molar roots and the mandibular canal [10]. However, cone-beam computed tomography (CBCT) offers greater accuracy by providing detailed three-dimensional cross-sectional images, allowing clinicians to better visualize the spatial relationship between the molar roots and the canal throughout various stages of eruption [11,12].

## 2. Materials and Methods

This study complies with the Declaration of Helsinki and was approved for publication by the ethics committee of the Municipal Emergency Hospital, Timisoara (Romania) where the study was conducted (approval code: E-488; approval date: 7 February 2025).

The study is retrospective observational and involved analyzing a database containing a total of 523 mandibular CBCTs. Inclusion criteria were the presence of one or both lower third molars with fully formed roots. Exclusion criteria were represented by the presence of the lower third molar in the form of root remnants, extrusion of the lower third molar due to the lack of the antagonist, mesial tipping due to the lack of the lower second molar, supernumerary teeth in the region of interest, and the presence of mandibular tumors located in the lower third molar area. The study had no gender or age restrictions. Considering the inclusion and exclusion criteria, a total of 253 lower third molars were included in the study. In 67 cases, the presence of both lower third molars was recorded, while in 119 cases, the presence of a single lower third molar was recorded.

CBCT scans included in this study were obtained as part of routine diagnostic evaluations at our institution and were not limited to third molar assessment alone. In addition to preoperative planning for lower third molars, these scans were also performed for the investigation of other maxillofacial pathologies. As such, the sample was not restricted to symptomatic or high-risk patients, thereby helping to minimize selection bias.

CBCT images were processed using OnDemand3D Communicator™ software, version 1.0 (Cybermed Inc., Daejeon, Republic of Korea). The images were analyzed by two oral and maxillofacial surgeons with extensive experience in 3D imaging.

**Figure 1:**
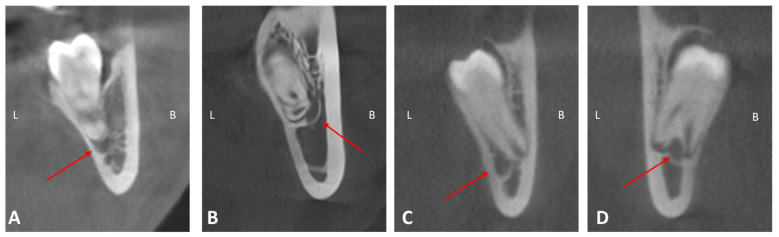
CBCT images illustrating the positional relationship of the mandibular canal relative to the mandibular third molar, classified into four types: (**A**) class I—apical position; (**B**) class II—buccal position; (**C**) class III—lingual position; (**D**) class IV—between the roots. Red arrows indicate the mandibular canal.

**Figure 2:**
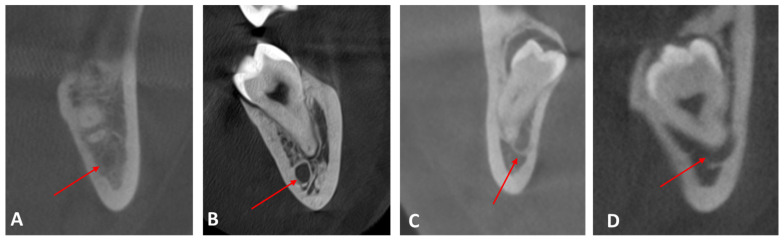
CBCT images demonstrating different contact relationships between the mandibular third molar and the mandibular canal: (**A**) No contact; (**B**) Contact with a complete white line; (**C**) Contact with a defective white line; (**D**) Penetration of the mandibular canal. Red arrows indicate the mandibular canal.

The 3D positional relationship of the mandibular canal relative to the lower third molar was determined by classifying it into the following classes: class I (apical position), class II (buccal position), class III (lingual position), and class IV (between the roots) (Figure 1). In all four classes above, the contact relationship between the third molar and the mandibular canal was also analyzed as follows: no contact, contact with a complete white line, contact with a defective white line, and penetration of the mandibular canal (Figure 2). If there is no contact, an additional measurement was made, namely the distance measured in millimeters between the mandibular canal and the lower third molar. Distances were divided into ranges (0–2 mm, 2–4 mm, 4–6 mm, 6–8 mm, 8–10 mm, over 10 mm).

The data acquired from the CBCT analysis were recorded in Microsoft Excel and later transposed into SPSS software v30 for statistical processing. Two types of statistical analysis were performed, descriptive and contingency analysis.

The descriptive analysis presents the distribution of cases according to the criteria of sex, age, location of the lower third molar (right or left), position of the mandibular canal, contact of the mandibular canal with the apices of the third molar, and the distance between the mandibular canal and the apices of the lower third molar.

The analysis of the contingency tables was performed using the Chi-Square Likelihood Ratio test and aimed to answer two hypotheses formulated before the start of the study. The first of the two hypotheses refers to the risk of injury to the alveolar nerve that is found inside the mandibular canal during the extraction of the lower third molar. We considered the lowest level of risk in the case where the mandibular canal has no contact with the apices of the third molar and that the risk increases progressively, being at the highest level in the case where we have a penetration of the mandibular canal by the apices of the third molar. Taking this consideration into account, we desired to establish in which of the 4 classes of position of the mandibular canal is the risk of injury to the inferior alveolar nerve higher. The second hypothesis also refers to the risk of injury to the inferior alveolar nerve during extraction, but only in cases without contact between the mandibular canal and the apices of the lower third molars. Considering that the risk of injury progressively decreases with increasing distance between the mandibular canal and the apices, we desired to establish in which of the position classes this risk is higher.

## 3. Results

Of the total of 253 lower third molars that were included in the study, 54.9% were found in women (*n* = 139) and 45.1% in men (*n* = 114). This distribution suggests a slight predominance of women in the sample.

The mean age was M = 37.82 years, with a median of 37 years, and the most frequent value (mode) was also 37 years. The age of the participants varied from a minimum of 14 years to a maximum of 68 years, with a standard deviation of SD = 12.121, indicating moderate variation.

**Figure 3:**
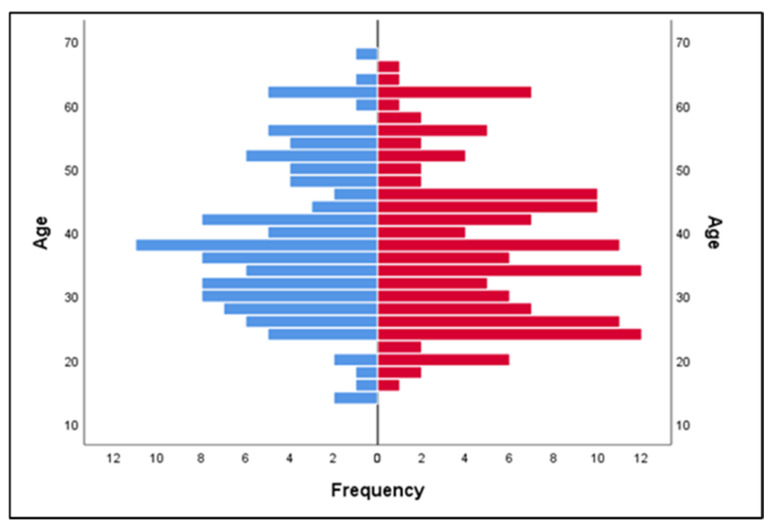
Distribution of ages of participants by gender; men are represented in blue and women in red.

Figure 3 illustrates the distribution of ages of participants by gender, highlighting the different age proportions for men and women. The graph shows a distribution with a frequency concentration around the ages of 30–40. Men (represented in blue) and women (represented in red) show a similar distribution, but there are slight differences in certain age groups, suggesting a slight variability in the age structure by gender.

Of the 253 third molars included in the study, 49.4% (*n* = 125) were lower right third molars and 50.6% (*n* = 128) were lower left third molars. The proportions reflect a balanced distribution between the two categories, with a slight predominance of the lower left third molar.

Table 1 presents the distribution according to the position of the mandibular canal, classified into four categories: class I, class II, class III, and class IV. According to the table, the majority of lower third molars 70.8% (*n* = 179) are found in class I, where the mandibular canal is located on the apical side. Class II, where the mandibular canal is located on the buccal side, includes 17.4% of the lower third molars (*n* = 44), and class III, where the canal is located on the lingual side, includes 8.3% (*n* = 21). Finally, class IV, where the canal is located between the roots, represents only 3.6% of the lower third molars (*n* = 9).

Table 2 show the distribution according to the contact between the third molar and the mandibular canal. The results show that the majority of cases do not present contact, this being observed in 66.0% of cases (*n* = 167). A smaller percentage, 22.1% (*n* = 56), present contact with a defective white line, while 10.7% (*n* = 27) present contact with complete white line. Finally, in only 1.2% of the cases (*n* = 3), penetration of the mandibular canal was reported.

**Figure 4:**
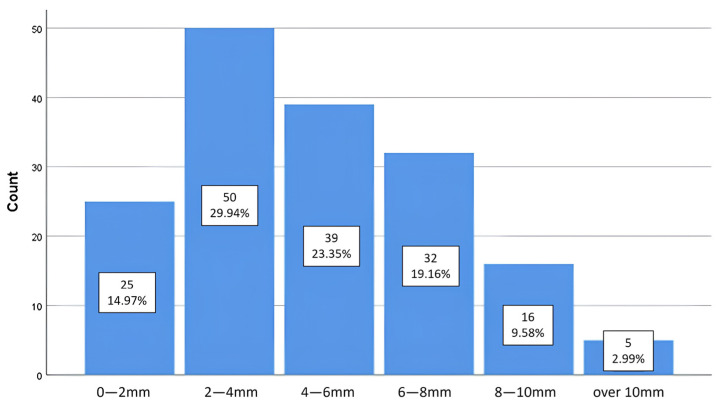
Distribution of cases by distance criterion.

Of the total of 253 lower third molars included in the study, 66.0% (*n* = 167) have valid values of the distance variable, namely those in the no contact category (Table 3). Among the valid cases, the most frequent interval is 2–4 mm, representing 29.94% (*n* = 50) of the total sample. The next most frequent interval is 4–6 mm, with 23.35% (*n* = 39), followed by the 6–8 mm interval, which represents 19.16% (*n* = 32). The interval with the smallest distance represents 14.97% (*n* = 25). The last intervals, 8–10 mm and over 10 mm, are the least frequent, with 9.58% (*n* = 15) and 2.99% (*n* = 5), respectively (Figure 4).

For the contingency analysis between mandibular canal position and contact type, the Likelihood Ratio test was used to examine the association between the variables (Table 4).

The results of the Likelihood Ratio test show a significant association between mandibular canal position and contact type (G^2^ = 65.548, *p* < 0.001). This indicates that there is a low probability that the observed distribution of cases is random, which supports an association between canal position and contact type.

Given the high value of the Likelihood Ratio test and its statistical significance (*p* < 0.001), the results suggest that it is very unlikely that the observed distribution between mandibular canal position and contact type is random. Thus, the existence of a strong association between the two variables is confirmed.

Specifically, the Likelihood Ratio test indicates significant differences in the distribution of contact types for the different mandibular canal positions, confirming that this association cannot be explained by chance.

The adjusted residuals (AR) from the contingency table analysis offer insight into which contact types differ significantly from expected frequencies across canal positions. Values with an absolute AR greater than 2 indicate statistically meaningful deviations. The analysis demonstrated a strong association between canal position and contact type. Class I (apical) was significantly associated with the absence of contact, suggesting a lower likelihood of nerve involvement. In contrast, Classes III (lingual) and IV (interradicular) exhibited higher-than-expected frequencies of contact with a defective white line and canal penetration, indicating elevated surgical risk. Class II (buccal) showed no significant deviations, with distributions consistent with expected values. These findings underscore the influence of canal position on contact risk and the importance of detailed radiographic evaluation in surgical planning.

**Figure 5:**
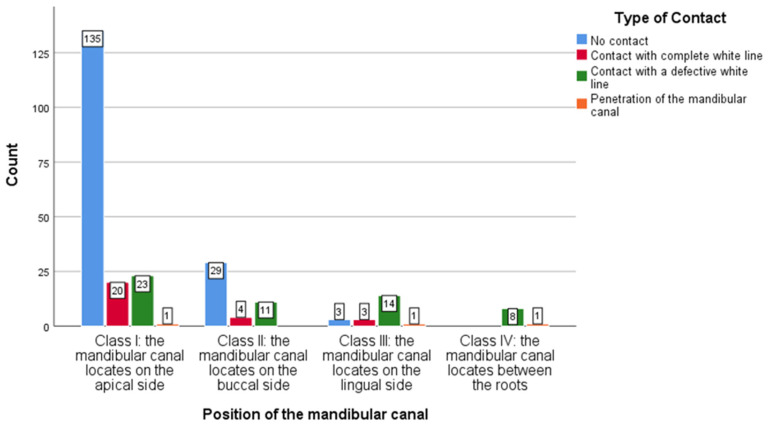
Distribution of cases from different types of contact according to the position of the mandibular canal.

The statistical analysis performed indicates significant differences in the distribution of contact types depending on the position of the mandibular canal, as can be seen in Figure 5.

Likelihood Ratio test was used for contingency analysis examining the relationship between mandibular canal position and apex–canal distance in non-contact cases. In this situation, the Likelihood Ratio test is significant (*p* = 0.046), suggesting that there is a significant association between the position of the mandibular canal and the distance between the apex and the canal. Adjusted residuals highlight significant deviations between observed and expected frequencies for certain positions and distance intervals (Table 5).

The adjusted residual analysis showed meaningful variations in the distance between the mandibular canal and third molar roots, depending on canal position. In Class I (apical), there were fewer cases than expected in the 2–4 mm range and more cases than expected in the 6–8 mm range, indicating a tendency for greater separation and reduced risk. In Class II (buccal), fewer cases than expected were found in the 6–8 mm range, suggesting closer proximity in this group. Class III (lingual) showed no significant deviations, meaning the distribution of distances matched statistical expectations. These findings highlight that canal position influences how close the roots are to the canal, which is important for assessing the risk of nerve injury—even when no direct contact is seen.

**Figure 6:**
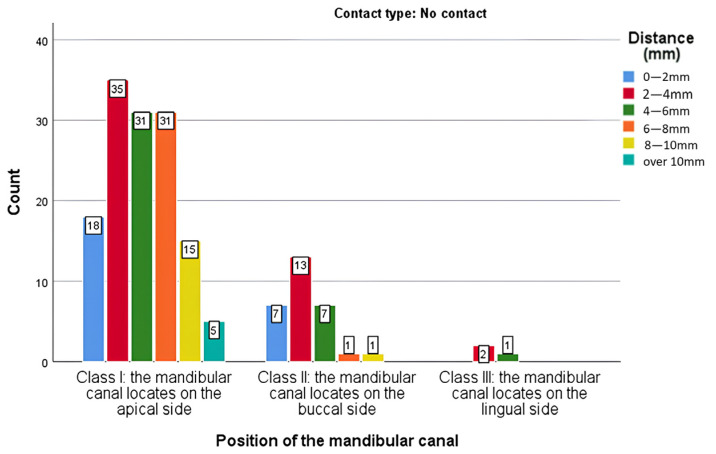
Distribution of cases from different distance ranges according to the position of the mandibular canal.

The results of the analysis suggest that, in general, there is a significant association between the position of the mandibular canal and the distance between the apex and the canal, according to the Likelihood Ratio test. The analysis of the adjusted residuals shows that certain distances may be more common for specific positions. The analysis of the position of the mandibular canal against the distances to adjacent structures highlights significant observations for Class I and Class II (Figure 6).

## 4. Discussion

Lower third molar extraction is the most commonly performed oral surgery procedure. Complications during or following lower third molar extraction are more common than with any other tooth extraction. This is due to the location of the tooth near nervous (inferior alveolar nerve, lingual nerve), vascular (inferior alveolar artery, facial artery), or muscular (masticatory muscle) anatomical structures. Also, the posterior location of the lower third molar and the increased bone density at that level are factors that favor the occurrence of complications [13,14,15,16,17,18].

The most common complications resulting from lower third molar extraction are postoperative and are mainly represented by inflammatory complications such as alveolitis, edema, trismus, and pain [19,20].

Intraoperative complications associated with the extraction of the lower third molar can be represented by injuries to nearby anatomical elements such as the inferior alveolar artery or inferior alveolar nerve and lingual nerve. These are manifested by serious intraoperative bleeding in the case of injury to the inferior alveolar artery and by sensory alterations in the case of injury to nerve structures [19,21,22]. These types of complications, which involve injury to the inferior alveolar nerve or lingual nerve, are encountered especially in cases where extensive osteotomy is necessary but also in cases where the extraction was performed under general anesthesia [23,24].

Any injury to the inferior alveolar nerve, located in the mandibular canal, can lead to paresthesia in the areas of distribution of this nerve. In most cases, full recovery occurs between 6–8 weeks after the trauma, although it can take up to 24 months. If the paresthesia does not completely resolve within about 2 months, the likelihood of a permanent deficit increases significantly [25].

OPG has long been the most widely used type of 2D radiography in third molar surgery. It is used for diagnosis, surgical planning, and anticipation of possible intra- or postoperative complications, the most important of which are injury to the inferior alveolar nerve or inferior alveolar artery [12,26,27]. OPG has the advantage of being an accessible investigation that visualizes multiple anatomical elements of importance in dental practice. It offers good visibility, especially in the posterior mandibular area [28].

Nevertheless, OPG has several drawbacks, including low image resolution, anatomical noise, overlapping structures, geometric distortion, and the presence of phantom images [11]. Therefore, the precise anatomical relationship between mandibular third molars and the inferior alveolar nerve cannot be accurately determined using this type of investigation. Although, due to limited socioeconomic accessibility and higher radiation exposure, CBCT is not a routine investigation; it provides a detailed three-dimensional visualization of the region, facilitating the highlighting of the relationship of the third molar root with the mandibular canal [12].

Studies in the literature that follow the complication rate in third molar surgery in situations where CBCT was used preoperatively compared to OPG do not show a significant decrease in the rate of inferior alveolar nerve injury but highlight its advantages in predicting this risk [29,30,31,32].

Other studies reinforce the clinical importance of understanding the three-dimensional anatomical relationship between the mandibular third molar and the mandibular canal, particularly in the context of preventing inferior alveolar nerve (IAN) injury during surgical extraction. These studies utilize CBCT, highlighting its superior diagnostic capabilities compared to traditional panoramic imaging in evaluating the spatial proximity and potential contact between the mandibular canal and the roots of third molars [9].

Gu et al. (2018) and Chaudhary et al. (2020) independently confirmed that lingual positioning of the mandibular canal is significantly associated with an increased risk of direct contact with the third molar roots and potential IAN injury [33,34]. Notably, both studies showed that lingually positioned canals had a markedly higher prevalence of canal contact or penetration, underscoring the need for careful preoperative risk assessment in such cases [33,34].

Complementing these findings, the study by Ge et al. (2016) [35] examined the buccolingual alveolar bone thickness surrounding impacted mandibular third molars. The results demonstrated that reduced lingual bone thickness was significantly associated with a higher likelihood of third molar proximity to the mandibular canal, potentially increasing the complexity of surgical extraction and the risk of nerve injury. This morphometric data supports the increased vulnerability of lingual canal positioning and underscores the need to account for alveolar bone topography in preoperative assessments [35].

The present study provides a comprehensive assessment of the three-dimensional relationship between the mandibular canal and the lower third molar using cone-beam computed tomography (CBCT). The findings confirm that the anatomical position of the mandibular canal plays a critical role in determining the risk of inferior alveolar nerve (IAN) injury during third molar extraction.

This study confirms that the anatomical position of the mandibular canal significantly influences the risk of inferior alveolar nerve (IAN) injury during third molar extraction. Consistent with prior research [33,34,35], lingual (Class III) and interradicular (Class IV) positions demonstrated the highest likelihood of canal contact or penetration, necessitating heightened caution during surgery. However, our findings also reveal two notable differences. First, the apical position (Class I) was the most commonly observed in our study, accounting for 70.8% of cases—higher than in previous reports, such as Gu et al. (2018) [33], where apical positioning was closer to 60%. Second, even in the absence of direct radiographic contact, risk remains—particularly for buccal (Class II) canals with apex–canal distances under 4 mm. While apical canals (Class I) were most frequent and typically associated with safer distances, the variation observed across all canal positions underscores the need for individualized preoperative assessment.

CBCT enables detailed visualization of these positional relationships, supporting more informed surgical decisions. Especially in high-risk scenarios—such as Class III and IV positions—CBCT findings may justify alternative techniques like coronectomy to minimize nerve damage.

Although this was primarily a radiographic study, several patients later underwent third molar extraction at our clinic. In three cases involving lingual (Class III) or interradicular (Class IV) canal positions with contact or penetration, transient IAN paresthesia occurred, resolving within 8–12 weeks. No complications were observed in apical (Class I) cases with canal distances over 4 mm. These clinical observations support our findings and highlight the surgical relevance of CBCT assessment.

However, the study has limitations. It is retrospective and lacks comprehensive postoperative data for all included patients. As such, clinical outcomes could not be statistically correlated with radiographic findings across the full sample. Future prospective studies with standardized follow-up would be valuable for validating the predictive value of canal position and apex–canal distance regarding surgical complications.

## 5. Conclusions

This study highlights the significance of cone-beam computed tomography (CBCT) in assessing the anatomical relationship between the mandibular canal and lower third molars. The apical canal position was most common and associated with the lowest risk of inferior alveolar nerve (IAN) injury. In contrast, lingual and interradicular positions showed higher rates of canal contact and penetration, indicating greater surgical risk.

Even in no-contact cases, a short apex–canal distance—particularly in buccal positions (Class II)—suggests residual risk. Statistical analysis confirmed significant associations between canal position, contact type, and proximity.

These findings support the routine use of CBCT for accurate risk assessment and surgical planning, especially in high-risk anatomical configurations. Careful evaluation can guide the choice of safer techniques and help prevent IAN injury during third molar extraction.

## Figures and Tables

**Table 1:** Distribution according to the position of the mandibular canal.

	Frequency (no.)	Percentage (%)
Class I: the mandibular canal locates on the apical side	179	70.8
Class II: the mandibular canal locates on the buccal side	44	17.4
Class III: the mandibular canal locates on the lingual side	21	8.3
Class IV: the mandibular canal locates between the roots	9	3.6
Total	253	100.0

**Table 2:** Distribution according to contact.

	Frequency	Percent
No contact	167	66.0
Contact with complete white line	27	10.7
Contact with a defective white line	56	22.1
Penetration of the mandibular canal	3	1.2
Total	253	100.0

**Table 3:** Distance interval.

		Frequency	Percent
Valid	0–2 mm	25	9.9
	2–4 mm	50	19.8
	4–6 mm	39	15.4
	6–8 mm	32	12.6
	8–10 mm	16	6.3
	over 10 mm	5	2.0
	Total	167	66.0
Missing	System	86	34.0
Total		253	100.0

**Table 4:** Contingency analysis between mandibular canal position and contact type.

			Contact				Total
			No Contact	Contact with Complete White Line	Contact with a Defective White Line	Penetration of the Mandibular Canal	
Position of the mandibular canal	Class I: the mandibular canal is located on the apical side	Count	135	20	23	1	179
		Expected count	118.2	19.1	39.6	2.1	179.0
		% within position of the mandibular canal	75.4%	11.2%	12.8%	0.6%	100.0%
		% within contact	80.8%	74.1%	41.1%	33.3%	70.8%
		Adjusted residual	4.9	0.4	−5.5	−1.4	
	Class II: the mandibular canal is located on the buccal side	Count	29	4	11	0	44
		Expected count	29.0	4.7	9.7	0.5	44.0
		% within position of the mandibular canal	65.9%	9.1%	25.0%	0.0%	100.0%
		% within contact	17.4%	14.8%	19.6%	0.0%	17.4%
		Adjusted residual	0.0	−0.4	0.5	−0.8	
	Class III: the mandibular canal is located on the lingual side	Count	3	3	14	1	21
		Expected count	13.9	2.2	4.6	0.2	21.0
		% within position of the mandibular canal	14.3%	14.3%	66.7%	4.8%	100.0%
		% within contact	1.8%	11.1%	25.0%	33.3%	8.3%
		Adjusted residual	−5.2	0.6	5.1	1.6	
	Class IV: the mandibular canal is located between the roots	Count	0	0	8	1	9
		Expected count	5.9	1.0	2.0	0.1	9.0
		% within position of the mandibular canal	0.0%	0.0%	88.9%	11.1%	100.0%
		% within contact	0.0%	0.0%	14.3%	33.3%	3.6%
		Adjusted residual	−4.3	−1.1	4.9	2.8	
Total		Count	167	27	56	3	253
		Expected count	167.0	27.0	56.0	3.0	253.0
		% within position of the mandibular canal	66.0%	10.7%	22.1%	1.2%	100.0%
		% within contact	100.0%	100.0%	100.0%	100.0%	100.0%

**Table 5:** Position of the mandibular canal in relation to the distance between the apex and the canal (no-contact cases).

			Distance						Total
			0–2 mm	2–4 mm	4–6 mm	6–8 mm	8–10 mm	Over 10 mm	
Position of the mandibular canal	Class I	Count	18	35	31	31	15	5	135
		Expected count	20.2	40.4	31.5	25.9	12.9	4.0	135.0
		% within position of the mandibular canal	13.3%	25.9%	23.0%	23.0%	11.1%	3.7%	100.0%
		% within distance (mm)	72.0%	70.0%	79.5%	96.9%	93.8%	100.0%	80.8%
		Adjusted residual	−1.2	−2.3	−0.2	2.6	1.4	1.1	
	Class II	Count	7	13	7	1	1	0	29
		Expected count	4.3	8.7	6.8	5.6	2.8	0.9	29.0
		% within position of the mandibular canal	24.1%	44.8%	24.1%	3.4%	3.4%	0.0%	100.0%
		% within distance (mm)	28.0%	26.0%	17.9%	3.1%	6.3%	0.0%	17.4%
		Adjusted residual	1.5	1.9	0.1	−2.4	−1.2	−1.0	
	Class III	Count	0	2	1	0	0	0	3
		Expected count	0.4	0.9	0.7	0.6	0.3	0.1	3.0
		% within position of the mandibular canal	0.0%	66.7%	33.3%	0.0%	0.0%	0.0%	100.0%
		% within distance (mm)	0.0%	4.0%	2.6%	0.0%	0.0%	0.0%	1.8%
		Adjusted residual	−0.7	1.4	0.4	−0.9	−0.6	−0.3	
Total		Count	25	50	39	32	16	5	167
		Expected count	25.0	50.0	39.0	32.0	16.0	5.0	167.0
		% within position of the mandibular canal	15.0%	29.9%	23.4%	19.2%	9.6%	3.0%	100.0%
		% within distance (mm)	100.0%	100.0%	100.0%	100.0%	100.0%	100.0%	100.0%

## Data Availability

The original contributions presented in this study are included in the article. Further inquiries can be directed to the corresponding authors.

## Abbreviations

The following abbreviations are used in this manuscript:

IAN	Inferior alveolar nerve
CBCT	Cone-beam computed tomography
OPG	Orthopantomograph
AR	Adjusted residuals
